# Nuclear spin-hyperpolarization generated in a flavoprotein under illumination: experimental field-dependence and theoretical level crossing analysis

**DOI:** 10.1038/s41598-019-54671-4

**Published:** 2019-12-05

**Authors:** Yonghong Ding, Alexey S. Kiryutin, Alexandra V. Yurkovskaya, Denis V. Sosnovsky, Renad Z. Sagdeev, Saskia Bannister, Tilman Kottke, Rajiv K. Kar, Igor Schapiro, Konstantin L. Ivanov, Jörg Matysik

**Affiliations:** 1 Institut für Analytische Chemie, Universität Leipzig, Linnéstr. 3, 04103 Leipzig, Germany; 2 International Tomography Center, Siberian Branch of Russian Academy of Sciences, Institutskaya, 3а, Novosibirsk, 630090 Russia; 3 Novosibirsk State University, Pirogova 1, Novosibirsk, 630090 Russia; 4 Physical and Biophysical Chemistry, Bielefeld University, Universitätsstr. 25, 33615 Bielefeld, Germany; 5 Fritz Haber Center for Molecular Dynamics Research, Institute of Chemistry, The Hebrew University of Jerusalem, Jerusalem, 9190401 Israel

**Keywords:** Solution-state NMR, Molecular biophysics, Biological physics, Biophysical chemistry

## Abstract

The solid-state photo-chemically induced dynamic nuclear polarization (photo-CIDNP) effect generates non-equilibrium nuclear spin polarization in frozen electron-transfer proteins upon illumination and radical-pair formation. The effect can be observed in various natural photosynthetic reaction center proteins using magic-angle spinning (MAS) nuclear magnetic resonance (NMR) spectroscopy, and in a flavin-binding light-oxygen-voltage (LOV) domain of the blue-light receptor phototropin. In the latter system, a functionally instrumental cysteine has been mutated to interrupt the natural cysteine-involving photochemistry allowing for an electron transfer from a more distant tryptophan to the excited flavin mononucleotide chromophore. We explored the solid-state photo-CIDNP effect and its mechanisms in phototropin-LOV1-C57S from the green alga *Chlamydomonas reinhardtii* by using field-cycling solution NMR. We observed the ^13^C and, to our knowledge, for the first time, ^15^N photo-CIDNP signals from phototropin-LOV1-C57S. Additionally, the ^1^H photo-CIDNP signals of residual water in the deuterated buffer of the protein were detected. The relative strengths of the photo-CIDNP effect from the three types of nuclei, ^1^H, ^13^C and ^15^N were measured in dependence of the magnetic field, showing their maximum polarizations at different magnetic fields. Theoretical level crossing analysis demonstrates that anisotropic mechanisms play the dominant role at high magnetic fields.

## Introduction

In general, nuclear magnetic resonance (NMR) methods are strongly limited by their intrinsically low sensitivity. Chemically induced dynamic nuclear polarization (CIDNP) is one of the hyperpolarization methods allowing for enhancement of intensity and sensitivity of NMR^1^. It is based on an effect generating transient non-Boltzmann distributed nuclear spin polarization, and it is detected by NMR as enhanced absorptive or emissive signal patterns. CIDNP effects mainly occur in photochemical reactions and, thus, are referred to as photo-CIDNP. In 1967, the liquid-state photo-CIDNP effect was discovered independently by Bargon and Fischer^2^ as well as by Ward and Lawler^3^. Two years later, it has been explained by the classical radical pair mechanism (RPM)^4,5^.

The restriction of studying samples with large molecular weight and high viscosity by solution NMR has led to the research on photo-CIDNP effects via solid-state magic-angle spinning (MAS) NMR. The solid-state photo-CIDNP effect was observed for the first time in frozen quinone-blocked bacterial photosynthetic reaction centers (RCs), where cyclic electron transfer was induced^6^. Since then, it has been observed in various natural RCs of bacteria^7–11^, diatoms^12,13^ and plants^14–18^.

Three different mechanisms have been proposed to explain the solid-state photo-CIDNP in photosynthetic systems^19^, namely, three-spin mixing (TSM)^20,21^, differential decay (DD)^22^, and differential relaxation (DR)^23^. For spin-1/2 nuclei, the two nuclear spin populations become polarized by these three mechanisms via two different decay pathways from singlet and triplet radical pair states. The mechanisms might run in parallel and are observed as a *net* contribution in a steady-state experiment^19,24,25^. At low fields, another window for the TSM is predicted^26^. Recently, the scheme has been re-interpreted in terms of electron-electron-nuclear level crossings and anti-crossings^27,28^ and regimes for optimal CIDNP formation have been determined.

With optimized parameters, the solid-state photo-CIDNP effect^7,19,29–32^ can enhance NMR signals by a factor of 100,000, allowing for a direct observation of the photo-chemical machineries of RCs in membrane preparations^33^, in whole cells^7,16^ and even in entire plants^18^. It can be used as a sensitive analytical tool to map electronic structures of photo-active cofactors^34^, given that the photo-CIDNP signal intensities are related to the local electron-spin density and thus the hyperfine coupling (HFC)^35^. The chemical shift of photo-CIDNP signals indicates the electronic environment of the ground-state electronic structure^8,36^. Also, time-resolved photo-CIDNP magic-angle spinning (MAS) NMR experiments were employed to probe the electronic spin-density distribution within the spin-correlated radical pair (SCRP) at atomic resolution during the progress of the reaction^37–39^ enabling an analysis of the kinetics of the solid-state photo-CIDNP effect and the underlying photo-cycle. In addition, the occurrence of the solid-state photo-CIDNP effect in liquid membrane samples demonstrates that sufficient orientation allows for its induction^33^.

**Figure 1:**
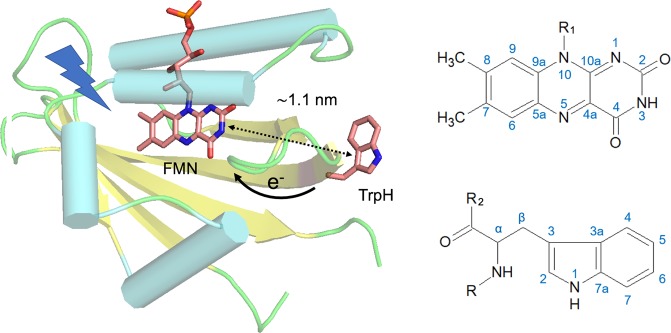
Crystal structure of wild-type phototropin-LOV1 in the dark with 1.9 Å resolution (PDB: 1N9L). The edge-to-edge distance of FMN to tryptophan is around 1.1 nm. Note that electron transfer from tryptophan (at position 98) to FMN after photo-excitation occurs only when the conserved cysteine close to FMN is mutated to serine or alanine. The IUPAC numbering of FMN and tryptophan are included.

So far, the photo-CIDNP effect has been observed in all photosynthetic organisms studied by solid-state NMR. In addition, a single non-photosynthetic system, the LOV (light-oxygen-voltage) domain of phototropin, was found to show the effect by both, MAS NMR under solid-state conditions^40,41^ and liquid-state NMR^42–44^. Phototropin is a flavin-binding photoreceptor, named after the phototropism as a directional growth of plants toward light stimuli^45,46^. It contains two similar photosensory LOV domains, each of which non-covalently binds flavin mononucleotide (FMN) as chromophore. The domains mediate signal transmission to the kinase domain dependent on incident light. The overall structure of these light-sensitive domains has also been found in other domains sensitive to oxygen and voltage, therefore the somewhat misleading designation LOV was assigned to the photosensitive proteins. The LOV domains absorb in the UV-A to blue-light region with maxima at around 380 nm and 450 nm. In the naturally occurring LOV domains, the FMN chromophore absorbs blue light upon illumination and rapidly forms a covalent adduct with a conserved adjacent cysteine; this state is considered the physiologically active state of a LOV domain^47^. The photoproduct returns to the dark state upon the breakage of the bond between the FMN and the protein. The adduct formation occurs within several microseconds, and it takes seconds (in some proteins hours) for the FMN-cysteinyl adduct to revert to the dark state^48,49^. A cysteine-to-serine mutation in LOV1 (here employed for the photo-CIDNP measurements) abolishes adduct formation and increases the lifetime of the photo-induced triplet state of FMN to 27 microseconds^49^, which is still lower than the lifetime of 200 microseconds in solution^50^. This prolonged lifetime enables an electron transfer from a nearby tryptophan (at position 98) to FMN upon illumination^51^ (Fig. 1). The edge-to-edge distance between the single tryptophan (Trp) and FMN is ~1.1 nm (~11 Å), the center-to-center distance is ~1.6 nm according to the crystal structures of phototropin-LOV domains^52,53^. Even though the formation of a SCRP [FMN^●−^—TrpH^●+^] has not been directly observed to date, the presence of the resulting, more stable neutral radicals of FMNH^●^ and Trp^●^ in cysteine-less LOV domains after irradiation were found by EPR and UV-vis spectroscopic studies^54,55^.

The photo-CIDNP mechanism of a cysteine-less LOV2 domain of phototropin from the oat *Avena sativa* has been explored previously by performing magnetic field-dependent ^13^C solution NMR experiments at 4.7–11.8 T^44^. In the present work, we report ^13^C, ^15^N and ^1^H photo-CIDNP effects over different magnetic field windows employing phototropin-LOV1-C57S from the green alga *Chlamydomonas reinhardtii*. Different isotope-labelling strategies enabled the observation of magnetic field-dependent ^13^C and ^15^N photo-CIDNP effects by field-cycling solution NMR. In addition, we observed a magnetic field dependence of hyperpolarized protons of residual water by measuring the protein in deuterated buffer. The magnetic field at which the polarization maximum is found, *B*_*max*_, is different for ^1^H, ^13^C and ^15^N NMR: *B*_*max*_(^1^H) < *B*_*max*_(^13^C) < *B*_*max*_(^15^N). Apparently, *B*_*max*_ is inversely proportional to the gyromagnetic ratio of the nuclei. As the level-crossing analysis shows, anisotropic mechanisms play the dominant role in these experiments.

## Results

### ^13^C photo-CIDNP effect observed in phototropin-LOV1-C57S

The photo-CIDNP effect has significantly enhanced the signals of tryptophan and FMN in the cysteine-devoid phototropin LOV1 and LOV2 domains. In addition to such signal enhancement, selective isotope labelling of FMN or tryptophan in the protein would allow for faster and more sensitive measurement of kinetics and magnetic field-dependence of the effect. A previous publication^44^ has investigated the cysteine-lacking LOV domain reconstituted with different ^13^C-enriched FMN isotopologs. In the present contribution, we isotopically labelled the electron donor tryptophan, in order to obtain a complete understanding of the pair of residues that generates the photo-CIDNP effect in the LOV domain. Phototropin-LOV1 has only one tryptophan residue and is suitable for the tryptophan labelling strategy.

**Figure 2:**
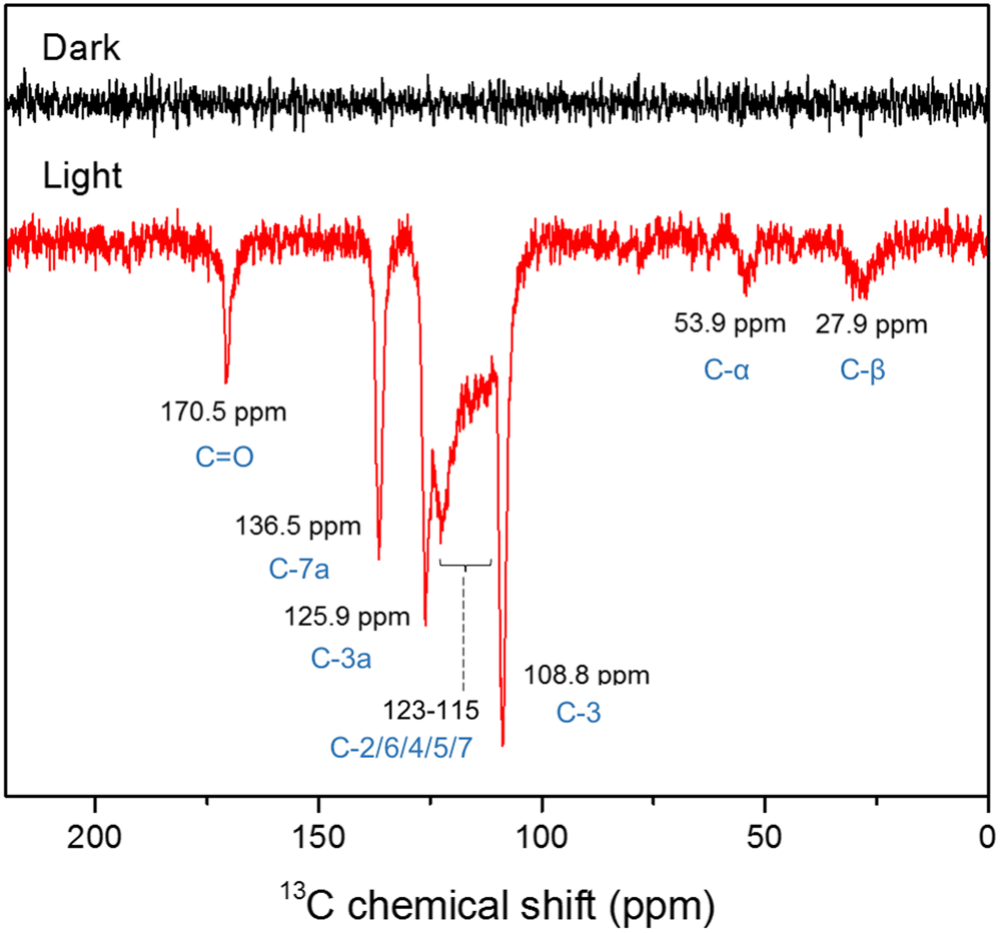
^13^C solution NMR spectra of phototropin-LOV1-C57S with u-^13^C enriched tryptophan under illumination (red) and in darkness (black) at 3.1 T, at each condition measured with 80 scans. Line broadening was set to 20 Hz. The photo-CIDNP signals were assigned to tryptophan carbons (blue).

The selective labelling of the electron donor tryptophan in the LOV domain by addition of u-^13^C labelled tryptophan was successful (Fig. 2). All ^13^C photo-CIDNP signals are emissive, opposite to the thermal polarization. The tentative assignment of the ^13^C photo-CIDNP signals was based on the biological magnetic resonance data bank and previous publications^44,56,57^. Since the tryptophan is fully ^13^C labelled, the various ^13^C-^13^C J-couplings split the signals into several lines, leading to a broad hump in the range of 115–126 ppm which renders the assignment of signals from C-2, C-4, C-5, C-6, and C-7 rather difficult. Future experiments might implement ^13^C-^13^C homonuclear decoupling or employ singly labelled indole or indole with multiple ^13^C separated from each other. At this stage, we chose the signal from tryptophan C-3 for investigating the magnetic field dependence of the photo-CIDNP effect.

### ^15^N photo-CIDNP effect observed in phototropin-LOV1-C57S

**Figure 3:**
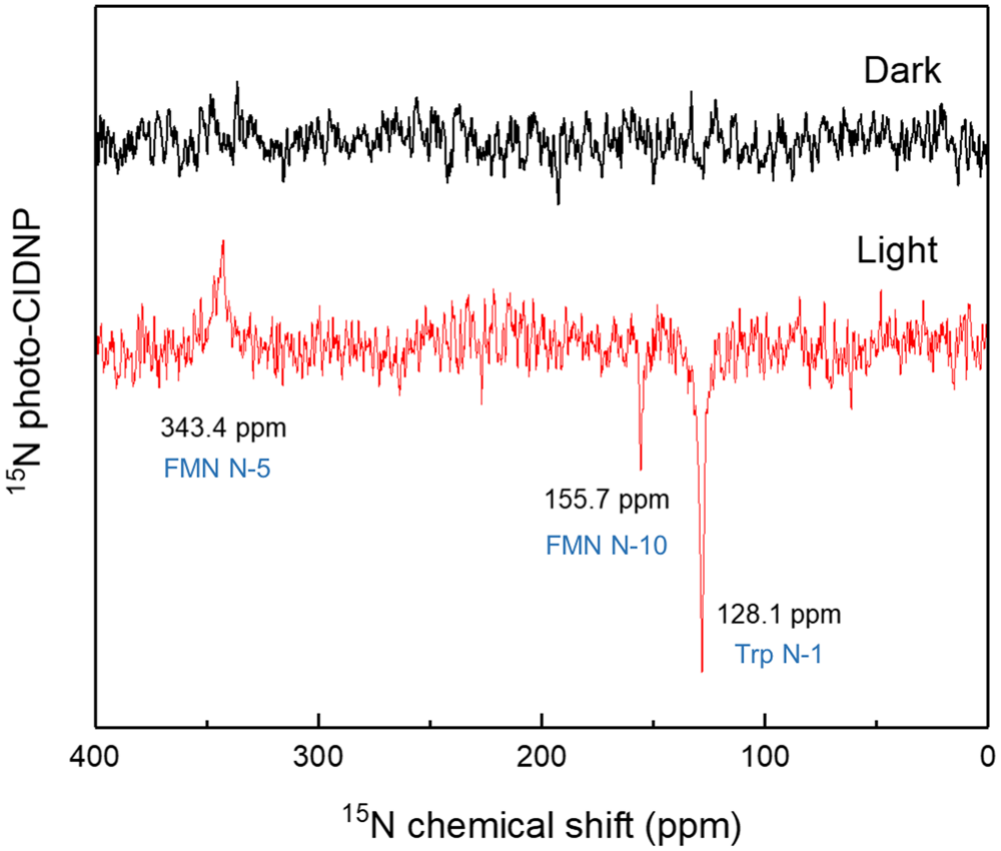
^15^N solution NMR spectrum of globally ^15^N labelled phototropin-LOV1-C57S measured at 5 T under illumination (red) and in darkness (black), each for 40 scans. Line broadening was set to 20 Hz for both spectra. Tentative assignment of several signals is provided in blue colour.

As for ^13^C, the system is expected to generate a photo-CIDNP effect for ^15^N and ^1^H if appropriate conditions are met. Labelling the protein globally by feeding ^15^NH_4_Cl has indeed allowed for observation of the ^15^N photo-CIDNP effect in solution NMR at 5 T (Fig. 3). To the best of our knowledge, this is the first report of a ^15^N photo-CIDNP effect from a cysteine-lacking LOV domain. The ^15^N photo-CIDNP signals are phased in reference to the external standard urea. The three light-induced signals appearing have different sign, hinting for the involvement of different mechanism running in parallel. The emissive ^15^N photo-CIDNP signal at 128.1 ppm is located in the range where the indole ^15^N is expected to appear^58,59^. Unequivocal assignment to the ^15^N at the tryptophan side chain is achieved by measuring the same protein labelled only with the ^15^N indole (Fig. S1). According to the TSM mechanism, the signal enhancement is related to the local electron spin density of the SCRP^35^. Previous theoretical analysis indicated that the N-10 and N-5 of the FMN anion radical carry significant hyperfine interactions in the FMN anion radical state compared to the other nitrogens of the FMN^60,61^. Hence according to the chemical shift^62,63^ and the calculated hyperfine interaction, we tentatively assign the absorptive signal appearing at 343.4 ppm to N-5 of FMN and the emissive one at 155.7 ppm to N-10 of FMN. In the present contribution, we focus on the intensity change of the ^15^N photo-CIDNP signal of indole nitrogen at 128.1 ppm at different magnetic fields. The sign of the light-induced hyperpolarization for the signal at 128.1 ppm is opposite to its thermal polarization.

### ^1^H photo-CIDNP effect of water from phototropin-LOV1-C57S in solution

**Figure 4:**
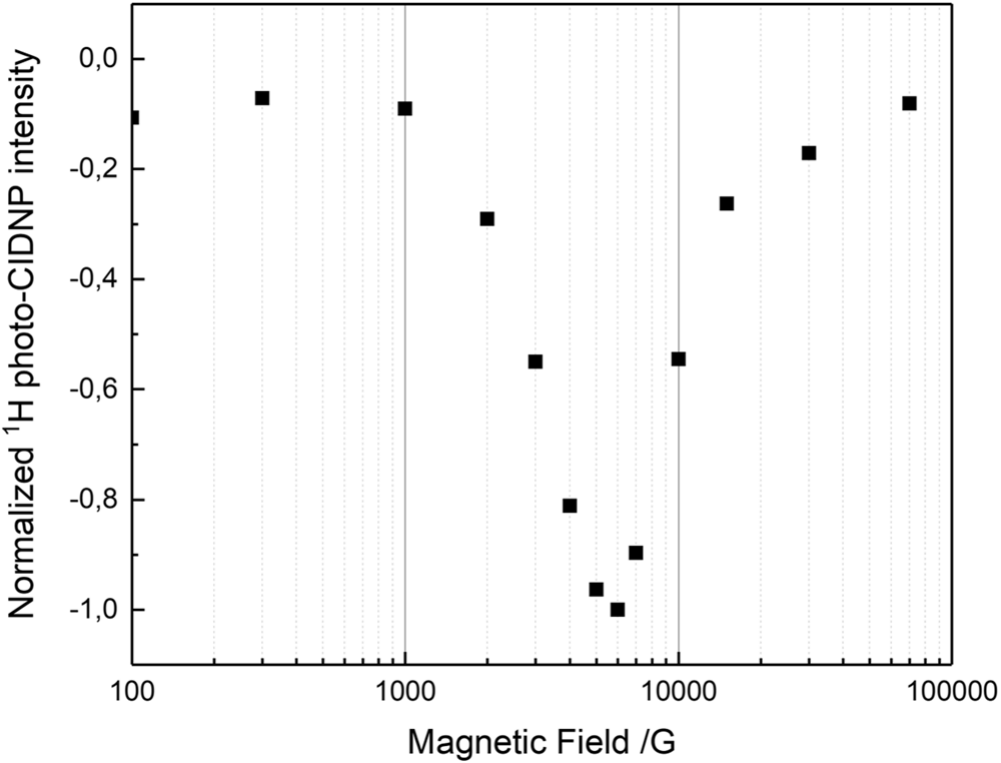
Magnetic field dependence of the ^1^H photo-CIDNP signal intensity of the water peak generated in the deuterated solution of phototropin-LOV1-C57S measured at magnetic fields ranging from 100 G (0.01 T) to 94000 G (9.4 T). At each magnetic field, two scans were performed in darkness and under illumination, respectively.

With the demonstration of ^13^C and ^15^N photo-CIDNP effects from LOV1-C57S, we extend our investigation to ^1^H photo-CIDNP. Using the protein with naturally abundant isotope distribution, the protein solution was exchanged with deuterated buffer beforehand to reduce the dominant water signal obtained in the ^1^H spectra. The sample was measured over a broad range of magnetic fields from 0.01 T up to 9.4 T. However, no obvious ^1^H polarization signals were observed in the chemical shift range in which protons of FMN or tryptophan are expected to appear (Fig. S2A), whereas there is a broad signal at around 4.7 ppm in the light-minus-dark intensity spectrum. We assigned the signal to residual water (HDO) in the system because of its chemical shift, line shape and dominating line strength. The signal is unlikely to result from protons on the protein or chromophore which should yield a sharper polarization signal. Following this observation, the signal at around 4.7 ppm was probed instead, using a pulse sequence without water suppression during the detection. Spectra under light and dark conditions are shown in Fig. S2B and the light-minus-dark differences are plotted against the applied magnetic fields (Fig. 4).

The ^1^H signal at around 4.7 ppm indeed shows a magnetic field-dependent polarization, i.e., a magnetic field effect (MFE) in the region of 0.1 T and 1.6 T (Fig. 4) with maximum polarization at around *B*_*max*_ = 0.6 T. The sign of light-induced ^1^H hyperpolarization is also opposite to its thermal polarization. Apparently, the magnetic field strength required for observation of the ^1^H polarization in the cysteine-lacking flavoprotein system appears to be too low for modern NMR spectrometers and therefore has not been reported so far. Thus, the application of a field-cycling system for the measurement of ^1^H photo-CIDNP is needed.

### Magnetic field dependence of ^13^C, ^15^N and ^1^H photo-CIDNP effects

**Figure 5:**
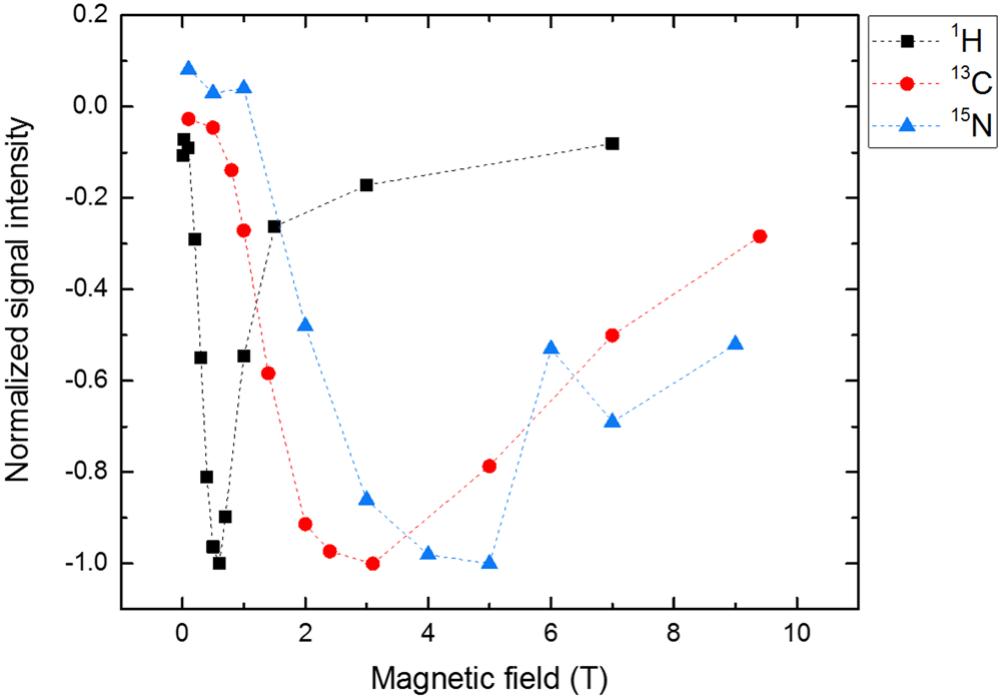
Comparison of magnetic field dependence of ^13^C (red), ^15^N (blue) photo-CIDNP signals generated by the tryptophan of phototropin-LOV1-C57S and ^1^H (black) from residual water in the deuterated solution of natural abundant protein. Since all the light-induced signals were emissive (negative), the extrema of the polarization curves were standardized to −1.0 and all other polarizations were normalized to their corresponding maxima.

Application of the field-cycling NMR method allowed a successful detection of the magnetic field dependence of ^13^C and ^15^N photo-CIDNP effects of phototropin-LOV1-C57S. The original NMR spectra are shown in the supporting information (Figs. S3 and S4). To compare the field-dependent enhancement of the signals of three nuclei ^13^C, ^15^N and ^1^H, we normalized all polarizations to their corresponding maxima (taken as unity). The signs of the light-induced hyperpolarization were always opposite to that of the Boltzmann polarization and thus indicated as negative (Fig. 5). Since thermal polarization is positive for protons and ^13^C and negative for ^15^N, this means that ^1^H and ^13^C CIDNP is negative, while ^15^N CIDNP is positive.

In a previous publication by Kothe *et al*.^44^, the magnetic field dependence measurement of ^13^C photo-CIDNP signals was performed at four magnetic fields 4.7 T, 7.1 T, 9.4 T and 11.8 T. Here we observe ^13^C photo-CIDNP signals at a different magnetic field window from 0.1T to 9.4 T with eleven magnetic fields measured. Our result shows for tryptophan C-3 a maximum of hyperpolarization at a magnetic field of 3 T. On the other hand, the maximum of hyperpolarization of tryptophan C-3 locates at 7.1 T in the publication of Kothe *et al*.^44^. The different results might relate to the difference of the two LOV domains of phototropin, which also were obtained from different species. In our case, the polarization of tryptophan C-3 at 7.1 T for phototropin-LOV1-C57S is far from the maximum because, judging from Fig. 5, the ^13^C polarizations are larger at 5 T, 3.1 T, 2.4 T and 2 T.

In addition, the following trend was found for the magnetic fields, at which maximal polarizations of the three types of nuclei occur: *B*_*max*_(^15^N) > *B*_*max*_(^13^C) > *B*_*max*_(^1^H), which might correlate to the γ-values of these nuclei (see below). The photo-CIDNP enhancement of ^1^H appears at a different magnetic field window than those of ^13^C and ^15^N. This difference in field dependence implies that ^1^H was not polarized as a consequence of polarized ^13^C or ^15^N from tryptophan or FMN. Since the protons in the environment of the SCRP are not expected to carry electron spin density implying that they are not the source of the hyperpolarization, it is reasonable to assume that the polarization was transferred from the SCRP to free protons in its environment by chemical exchange with polarized nitrogen atoms of tryptophan and FMN. Possibly, ^1^H photo-CIDNP was first present on protons of tryptophan and FMN and subsequently quickly distributed to the environment (residual protons).

### Theoretical modeling

To understand the mechanism of CIDNP formation and to recognize whether the polarization originates from isotropic or anisotropic spin interactions, we provide theoretical modelling of the field dependence of CIDNP. To this end, we present numerical calculations of CIDNP considering coherent spin mixing in the SCRP, comprising a system of 8 coupled spins, being the two electron spins, and six nuclei spins, which are pairs of ^1^H, ^13^C, and ^15^N nuclei. The Hamiltonian of the spin system is written as follows:

1 $$\hat{ {\mathcal H} }={\omega }_{1e}{\hat{S}}_{1z}+{\omega }_{2e}{\hat{S}}_{2z}-\mathop{\sum }\limits_{i=1}^{6}\,{\omega }_{N}^{(i)}{\hat{I}}_{iz}-{J}_{ex}[2({\hat{{\bf{S}}}}_{1}\cdot {\hat{{\bf{S}}}}_{2})+\frac{1}{2}]+{\hat{{\bf{S}}}}_{1}\hat{{\bf{D}}}{\hat{{\bf{S}}}}_{2}+\mathop{\sum }\limits_{i=1}^{6}({a}_{i}{\hat{S}}_{1z}{\hat{I}}_{iz}+{b}_{i}{\hat{S}}_{1z}{\hat{I}}_{ix})$$

Here **Ŝ**_1_and **Ŝ**_2_ are the electron spin operators of radical 1 (carrying *i* nuclei) and radical 2, and ***Î***_*i*_ is the spin operator of the *i*-th nucleus belonging to radical 1. We introduce the following interactions:

2 $${\omega }_{1e}={g}_{zz}^{(1)}{\mu }_{B}{B}_{0},\,{\omega }_{2e}={g}_{zz}^{(2)}{\mu }_{B}{B}_{0},\,{\omega }_{N}^{(i)}={g}_{N}^{(i)}{\mu }_{N}^{(i)}{B}_{0}$$

are the Zeeman interactions with the magnetic field *B*_0_, which are given by the corresponding *g*-factors of the electrons and nuclei and magnetons (the Bohr magneton for the electron and nuclear magnetons). For the electrons, we consider that the *g*-tensor is anisotropic and only take into account the *zz*-component of the *g*-tensor (other components are irrelevant in the high-field approximation), which is different for different molecular orientations: g_zz_ = g_xx_ sin^2^θ cos^2^φ + g_yy_ sin^2^θ sin^2^φ + g_zz_ cos^2^θ (here g_xx_, g_yy_, g_zz_ are the principal values of the *g*-tensor and angles θ and φ define orientation of the principal axes system with respect to the lab frame). The electron-electron coupling term comprises the exchange interaction of the strength *J*_*ex*_ and the dipolar interaction, which is expressed via the coupling tensor $\hat{{\bf{D}}}$. At high fields, the following terms are relevant

3 $${\hat{{\bf{S}}}}_{1}\hat{{\bf{D}}}{\hat{{\bf{S}}}}_{2}\approx \frac{1}{2}\cdot \frac{{D}_{0}}{{r}_{ee}^{3}}(1-3{\cos }^{2}\theta )(2{\hat{S}}_{1z}{\hat{S}}_{2z}-{\hat{S}}_{1x}{\hat{S}}_{2x}-{\hat{S}}_{1y}{\hat{S}}_{2y})$$

where *D*_0_ ≈ 1.9 mT × nm^3^ and *r*_*ee*_ is the distance between the radical centers and *θ* is the angle between the magnetic field and the vector connecting the radical centers. For each electron-nuclear HFC term, we take into account the secular coupling with the strength of *a*_*i*_ and pseudo-secular coupling of the strength *b*_*i*_. Hence, anisotropic interactions considered are the electron Zeeman interaction, electronic dipolar coupling and HFCs. We calculate CIDNP for only one single specific orientation because averaging over different orientations is time consuming. For the chosen orientation the vector connecting the radicals is ${{\bf{r}}}_{ee}=(\frac{2}{3},\,\frac{2}{3},\,\frac{1}{3})\times 1.6$ nm and the orientation of the principal axes of the *g*_2_-tensor is the same as the lab frame axes. We also assume that the *g*-tensor of the first radical is isotropic and equal to *g*_1_ = 2.0034 (this value was taken from Kopka, B. *et al*.^54^). Here, we do not average CIDNP over orientations for the sake of simplicity; in the calculation we assume isotropic *g*_2_ = 2.0026. We also assume that *J*_*ex*_ = −0.54 mT and *r*_*ee*_ = 1.6 nm, the distance of centers of the FMN and tryptophan in the LOV domain. HFC parameters are given below; in all cases we use the following reactivity constants of the radical pair: *k*_*S*_ = 0.01 ns^−1^ (singlet-state recombination rate), *k*_*T*_ = 0 (triplet-state recombination rate), *k*_*sc*_ = 10^−3^ ns^−1^ (spin-state independent reactivity). To evaluate the production of CIDNP, we assume that the radical pair is triplet-born and use the same method as previously^28^. All parameters used in the simulation are summarized in Table S2 in the SI. Here it is convenient to measure all interactions in the field units, since we look at the CIDNP field dependence.

Firstly, we present the CIDNP field dependence in the *anisotropic case* and take the following HFC parameters *a*_*H*1_ = 0.7 mT, *a*_*H*2_ = 0.5 mT, *a*_*C*1_ = 1.25 mT, *a*_*C*2_ = 1 mT, *a*_*N*1_ = 0.2 mT, *a*_*N*2_ = −0.1 mT, *b*_*H*1,2_ = 0.1*a*_*H*1,2_, *b*_*C*1,2_ = 2*a*_*C*1,2_, *b*_*N*1,2_ = 2.7*a*_*N*1,2_. Pseudo-secular couplings *b*_*i*_ were estimated from quantum chemical calculations as differences between the principal values of the corresponding HFC tensors. These calculations were performed on a truncated model of FMN, namely the lumiflavin, which was previously^64^ benchmarked by some of the present authors. Here we have studied lumiflavin in its radical anion (Lumi^●−^) form and the tryptophan residue in its radical cation (TrpH^●+^) form. The anisotropic spin dipole couplings for selected magnetic nuclei are summarized in Table S1.

**Figure 6:**
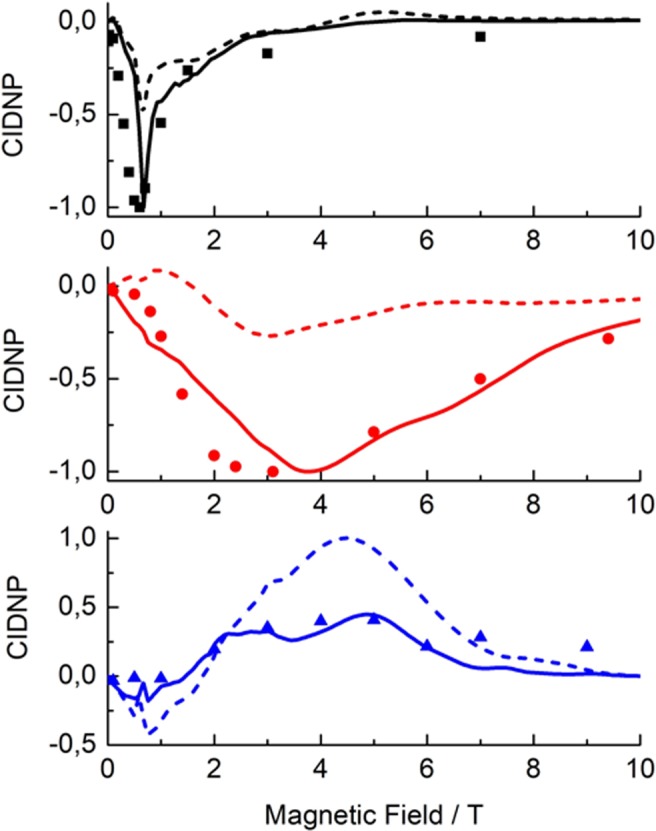
Comparison of the experimental (dots) and simulated (lines) CIDNP field dependencies for ^1^H (top), ^13^C (middle) and ^15^N (bottom) nuclei. In simulations, we take into account anisotropic spin interactions and consider an RP with two nuclei of each kind (altogether six nuclei). Solid and dashed fitting curves of the same color correspond to the polarization of the two nuclei of the same isotope species with different HFC constants. Simulation parameters are given in the text.

The results are shown in Fig. 6. Both curves in each subplot were normalized to the maximum value of polarization. In the upper subplot, polarization on the protons is presented, in the middle one on the carbon nuclei, in the lowest subplot on the nitrogen nuclei. CIDNP of ^1^H and ^13^C is *negative*, while ^15^N polarization is *positive*, which is consistent with the experimental observation. Solid and dashed curves of the same color correspond to the polarization of the two nuclei of the same isotope species with different HFC constants.

Hence, the calculation reproduces the experimental observation that CIDNP maxima are observed at different field strengths for the different nuclei. Such a behavior is expected when anisotropic interactions come into play and CIDNP is formed due to TSM, which depends on the electron-electron coupling and anisotropic HFC. The position of the maximum can be estimated from the expression |*ω*_*N*_ |≈ |*d|* = |−2*J*_*ex*_−*D|*. A more precise analysis suggests an interplay of several mechanisms, mainly TSM and DR. At different orientations, different mechanisms might dominate, so the magnetic field dependence of CIDNP after orientation averaging can be rather complex. Generally speaking, polarization of the solvent protons can occur for two reasons. One possibility is that tryptophan protons get polarized and subsequently take part in exchange with the solvent protons so that CIDNP is transferred to water. The second possibility is that solvent protons are directly coupled to the radicals by electron-nuclear dipole coupling, which enable CIDNP formation by anisotropic mechanisms. Presently, we are not able to reject any of these mechanisms, but we would like to mention that agreement with the experimental data is better if we use the HFC parameters for the protons of tryptophan. In any case, we reproduce the experimental observation that ^1^H CIDNP is formed at a significantly lower field as compared to the case of ^13^C nuclei.

**Figure 7:**
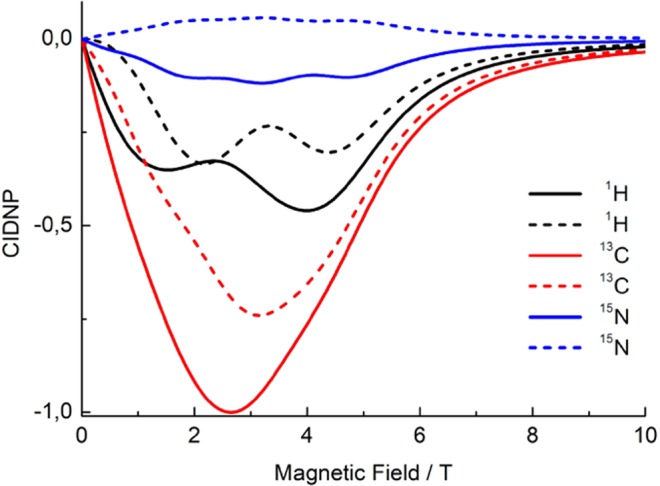
Comparison of simulated CIDNP field dependences for ^1^H (black), ^13^C (red) and ^15^N (blue) nuclei. In the simulations, we only take isotropic spin interactions into account and consider an RP with two nuclei of each kind (altogether six nuclei). Solid and dashed curves of the same color represent the polarization of the two nuclei of the same kind with different HFC constants. Simulation parameters (except for the pseudo-secular HFCs, which are taken zero) are the same as in Fig. 6.

In contrast, such a behavior is unexpected for isotropic mixing, where the value of *ω*_*N*_ does not affect the spin dynamics in the radical pair and CIDNP formation. To investigate whether the CIDNP formation efficiency does not depend on the nuclear *γ*-ratio in the case of isotropic spin mixing, we have run a simulation with exactly the same parameters except for the *b*_*i*_ values, which were set to zero (Fig. 7).

The features in the calculated CIDNP field dependencies are observed in the same field range for the different kinds of nuclei, which is typical for isotropic mixing where the only relevant parameter is the effective HFC of all nuclei, *a*_*eff*_. The positions of the features are determined by the matching condition: $|\Delta {\omega }_{e}|\approx |{a}_{eff}|$. Although additional features in the field-dependence of ^1^H and ^15^N CIDNP are present, caused by the mutual influence of different nuclei, all in all different nuclei behave similarly, in contrast to the anisotropic case.

## Discussion

The cysteine-devoid LOV domain of the biological blue-light receptor phototropin showed a photo-CIDNP effect, however, the mechanisms for it remained unclear. We isotopically labelled phototropin-LOV1-C57S and observed magnetic field dependent photo-CIDNP signals of three nuclei by taking advantages of field-cycling solution NMR. The results reveal that ^1^H, ^13^C, and ^15^N photo-CIDNP enhancement curves have different MFE maxima, *B*_*max*_, (Fig. 5) showing this trend: *B*_*max*_(^1^H) < *B*_*max*_(^13^C) < *B*_*max*_(^15^N), inversely proportional to their gyromagnetic ratio. The level crossing and level anti-crossing analysis of this experimental observation allows for better fitting of the magnetic field dependent data (Fig. 6), taking anisotropic interactions into account, while isotropic mixing taken as a mechanism leads to a contradictory result (Fig. 7). This implies that the photo-CIDNP effect occurring in this cysteine-lacking LOV domain is the result of solid-state mechanisms even though it has been measured in solution state. This conclusion is not surprising since previous experiments on liquid membrane samples have already demonstrated that the solid-state photo-CIDNP mechanisms do not require solid-state conditions to operate, but sufficient orientation on the NMR time scale^33^. The issue of motional averaging has been considered by Kothe *et al*.^44^ Generally speaking, if there is an interaction anisotropy of *H*_*aniso*_ it would be averaged by isotropic motions when the following condition is met: *H*_*aniso*_*τ*_*c*_ < 1 with *τ*_*c*_ being the motional correlation time, i.e., the time of molecular tumbling. In the opposite case, the interaction is not averaged out by motion. So, if we arrive at the condition *H*_*aniso*_*τ*_*c*_ > 1, anisotropic interactions need to be considered. Furthermore, in the situation where the radical-pair lifetime *t*_*RP*_ is very short, *H*_*aniso*_*τ*_*RP*_ < 1, we do not even need to consider variation of the interaction by motion (since averaging of *H*_*aniso*_ in the radical pair is anyway interrupted after the radical pair has recombined). Hence, due to the size of the protein, in the present study tumbling does not completely vanish the solid-state mechanisms. Motional averaging is taking place on the timescale longer than the radical-pair lifetime, meaning that anisotropic couplings, *g*-anisotropy, electron-electron coupling and hyperfine couplings are not averaged out in the SCRP. At the same time, we do not need to consider anisotropic nuclear couplings: for this reason, NMR spectra of the protein under study do not exhibit any traces of nuclear dipolar couplings or chemical shift anisotropy.

In addition, this contribution also displays the distribution of ^1^H polarization from the center of photochemistry, FMN or tryptophan, to the residual protons in deuterated solvent. The source of the polarization transfer can be chemical exchange of polarized protons on FMN or tryptophan, or direct coupling of the SCRP with environmental protons. Multiple mechanisms might be responsible and run in parallel for the photo-CIDNP effect occurring in cysteine-lacking LOV domains. In future work, we will aim to investigate the evolution of photo-CIDNP effect over time using laser-flash excitation with NMR detection. With this, the exact solid-state mechanisms might be disentangled. Furthermore, the experiments presented here were performed at relatively high field (tens of milli-Tesla and more). Therefore, our next goal will be to explore whether the protein system will show a photo-CIDNP effect at the magnetic field dimension of the earth, which is within the magnetic field range of the field shuttling system.

## Methods

### Isotope labelling and protein expression

^15^NH_4_Cl, [u-^13^C_8_] tryptophan and [^15^N] indole were purchased from Cambridge Isotope Laboratories, Inc. (Andover, MA, USA). The plasmid encoded phototropin-LOV1-C57S (amino acid residues 16–133, ~15 kDa) from *Chlamydomonas reinhardtii* carrying a 15 x His-tag at the N-terminus^65^. The heterologous overexpression in *E. coli* and purification at natural abundance was performed as previously reported^65^. Labelling of tryptophan followed the previous procedures by adding isotope-enriched indole or tryptophan as precursor to minimal medium during expression^66^. Likewise, the fully ^15^N-labelled protein was expressed by employing ^15^NH_4_Cl as a starting component in the minimal medium. The final protein solution was in phosphate buffer (300 mM NaCl, and 50 mM KH_2_PO_4_/K_2_HPO_4_, pH 8.0). For ^1^H NMR measurements, the protein solution was washed with deuterated phosphate buffer using centrifugal filters (the final solution may contain approximately 0.4% protons). The concentration of protein and chromophore occupancy was determined by UV-vis spectroscopy.

### Field-cycling of photo-CIDNP experiment by solution NMR

The ^13^C, ^15^N and ^1^H photo-CIDNP experiments were performed with a solution NMR spectrometer at 9.4 T (Bruker AVANCE III HD, furnished with an ultrashielded cryomagnet) equipped with a field-cycling setup. For the ^1^H photo-CIDNP experiment, naturally abundant phototropin-LOV1-C57S was employed; for the ^13^C and ^15^N photo-CIDNP experiments, corresponding isotope labelled samples were used. The preparation of the field-cycling experiment was mentioned in previous work^67^. Briefly, the sample was added to a standard 5-mm NMR tube, into which a solid light guide was inserted. As a light guide a quartz rod (20 cm in length and 4 mm in diameter) polished from both sides was used. It was inserted into the NMR sample tube and submerged into the solution. The position of the quartz rod was chosen so that its lower edge was 1–2 mm higher than the upper part of the receiving NMR coil. An LED chip with cupper radiator was attached to the upper end of the light guide. This design allowed us to illuminate the solution in a volume of NMR RF coil. The tube was mounted into the carriage of the shuttle device and moved to different magnetic fields by a rack-and-gear system. The position was controlled by a step motor which was synchronized with a pulse program. The maximum time for transfer of the sample from highest to lowest fields was about 0.5 s. For the protein studied here, a 440 nm LED (Chanzon, China) was employed with consumption power of 2 W (current 0.5 A and voltage 4 V) and the illumination time was optimized to 0.5 s. The temperature was set to 4 °C. Differences in shuttle times were compensated by adding a variable delay to ensure the same time offset after the illumination. To compensate the impact of photo-bleaching of the protein, a fresh aliquot from the same stock was measured at each magnetic field.

The pulse sequence used for the ^13^C and ^15^N photo-CIDNP experiments was, after the shuttle, pulse-acquire with WALTZ-16 proton decoupling. With regards to the ^1^H photo-CIDNP experiment on the protein, pre-saturation composite pulses were applied at the beginning, then the sample was shuttled to corresponding fields, illuminated for 0.5 s and returned, followed by excitation sculpting to suppress the water signal^68^. However, for measuring the light-induced polarization of the water signal, the same pulse sequence was used but excitation sculpting was replaced by pulse acquire sequence. The ^15^N NMR spectra and the ^13^C NMR spectra were phased to the external standard, a mixture of 0.1 M ^15^N urea and 0.1 M ^13^C methanol in DMSO. All ^15^N chemical shifts are relative to liquid ammonia and indirectly referenced to ^15^N signal of urea. For all photo-CIDNP experiments, the samples were first measured under darkness with the same number of scans and then measured under irradiation with blue light.

### Theoretical and quantum chemical calculations

Spin dynamics calculations were performed numerically using an algorithm developed for simulating CIDNP in photosynthetic reaction centers^28^. In this method, we used matrix algebra operations to compute polarization, i.e., inversion of super-operator matrices. The computer program has been modified for modeling CIDNP in a system of 8 coupled spins, two electrons and 6 spin-1/2 nuclei. Calculations were performed in the Liouville space and require dealing with super-matrices of a dimensionality 2^16^ × 2^16^. To implement the calculation method, we used a home-written Matlab code run on a desktop computer (2 CPU Intel Xeon at 2.4 GHz). Quantum chemical calculation of hyperfine coupling tensors were done using the Gaussian 09 program^69^. The geometries of the lumiflavin radical anion and the tryptophan radical cation were optimized using the B3LYP/aug-cc-pVDZ level of theory in the gas phase (Fig. S5). The minimum energy conformation was confirmed for both geometries with a Hessian calculation that resulted exclusively in real frequencies. The HFC tensor was calculated at the optimized geometry using the same level of theory.

## Supplementary information

Supplementary data
