# Supplementary material for: Nuclear spin-hyperpolarization generated in a flavoprotein under illumination: experimental field-dependence and theoretical level crossing analysis
Source: Sci Rep. 2019 Dec 5;9:18436. doi: 10.1038/s41598-019-54671-4 (PMC6895156; doi:10.1038/s41598-019-54671-4)
Supplement: Supplementary file 1 — Supplementary data [file 41598_2019_54671_MOESM1_ESM.pdf]

Supporting information
for:

Nuclear spin-hyperpolarization generated in a flavoprotein under illumination: experimental field-dependence and theoretical level crossing analysis

Yonghong Ding[1], Alexey S. Kiryutin[2,3], Alexandra V. Yurkovskaya[2,3], Denis V. Sosnovsky[2,3], Renad Z. Sagdeev[2,3], Saskia Bannister[4], Tilman Kottke[4], Rajiv K. Kar[5], Igor Schapiro[5], Konstantin L. Ivanov[2,3], Jörg Matysik[1*]

[1] Institut für Analytische Chemie, Universität Leipzig, Linnéstr. 3, 04103 Leipzig, Germany

[2] International Tomography Center, Siberian Branch of Russian Academy of Sciences, Institutskaya, 3a, Novosibirsk, 630090, Russia

[3] Novosibirsk State University, Pirogova 1, Novosibirsk, 630090, Russia

[4] Physical and Biophysical Chemistry, Bielefeld University, Universitätsstr. 25, 33615 Bielefeld, Germany

[5] Fritz Haber Center for Molecular Dynamics Research, Institute of Chemistry, The Hebrew University of Jerusalem, Jerusalem, 9190401, Israel

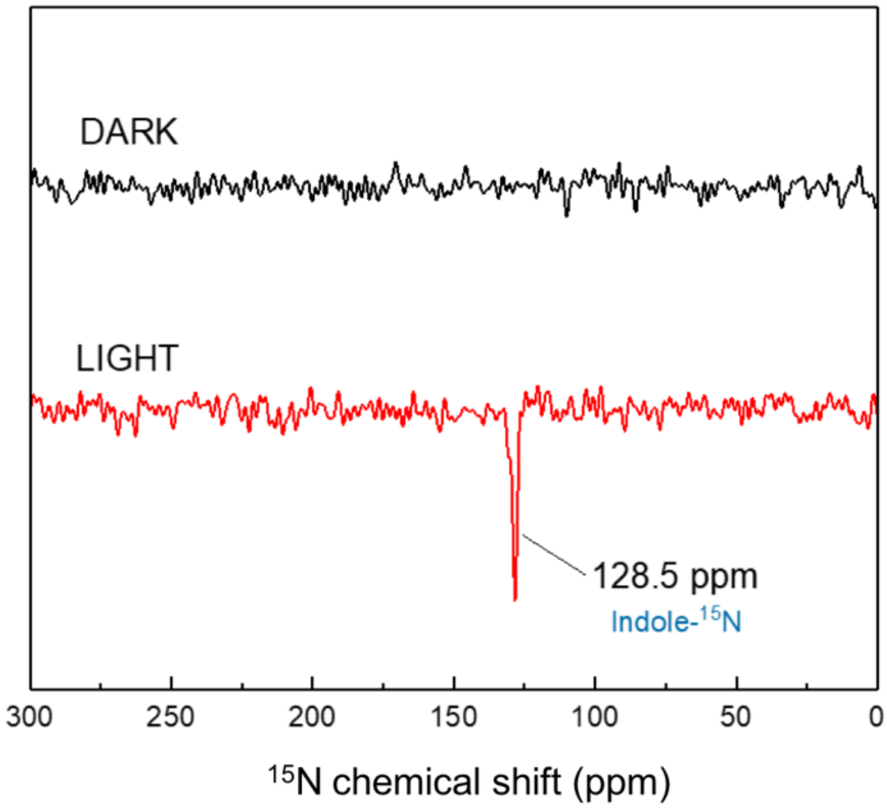

Figure S1. ^{15}N solution NMR spectrum of phototropin-LOV1-C57S with ^{15}N labelled side-chain of the single tryptophan, measured at 9.4 T for 40 scans under light (red) and dark (black) conditions, respectively; line broadening 20 Hz.

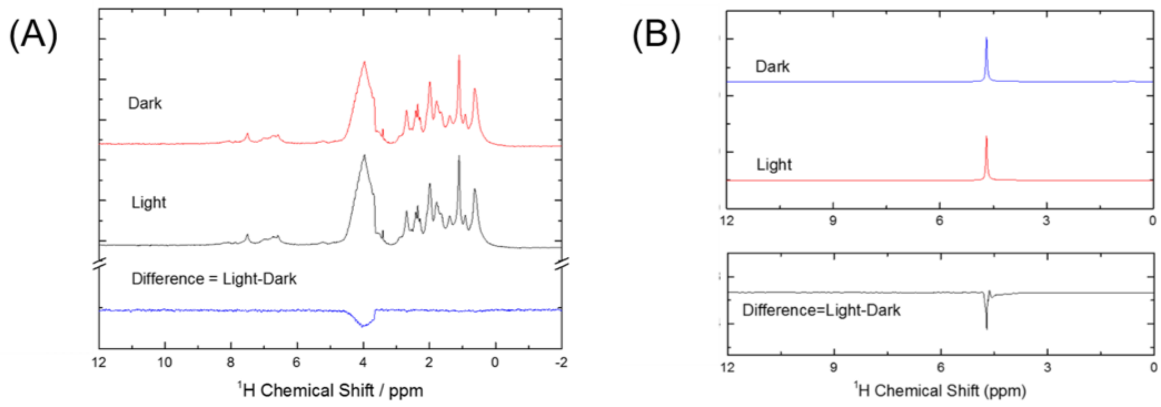

Figure S2. ^{1}H-NMR spectrum of phototropin-LOV1-C57S solution in deuterated buffer (with residual HDO), measured at 9.4 T for 32 scans under light and dark conditions with solvent suppression (A), and without solvent suppression (B); line broadening 5 Hz.

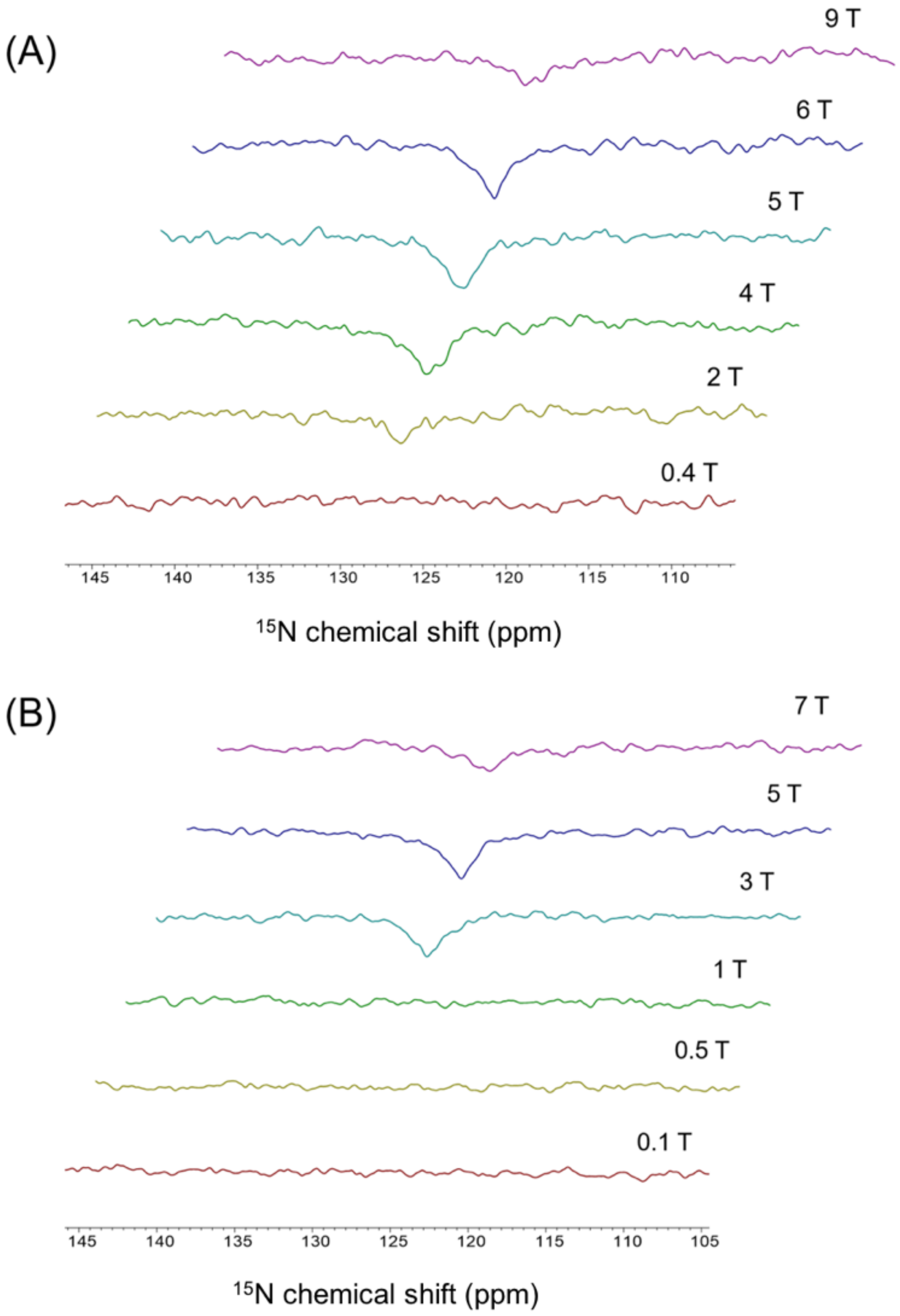

Figure S3. ^{15}N spectra of phototropin-LOV1-C57S measured under illumination at different magnetic fields using the field-cycling system based on a 9.4 T liquid-state NMR spectrometer. 40 scans were recorded for each magnetic field. The measurements were performed in two groups (A) and (B). In all spectra, line broadening

was set to 20 Hz; the hyperpolarized signal at ~127 ppm from each magnetic field was integrated and normalized to that of 5 T measured in the corresponding experiment; the normalized intensity was plotted against the magnetic field shown in Figure 5. We focused on the MFE of the signal at ~127 ppm. The MFE of the other signals is beyond the scope of this contribution and will be discussed elsewhere.

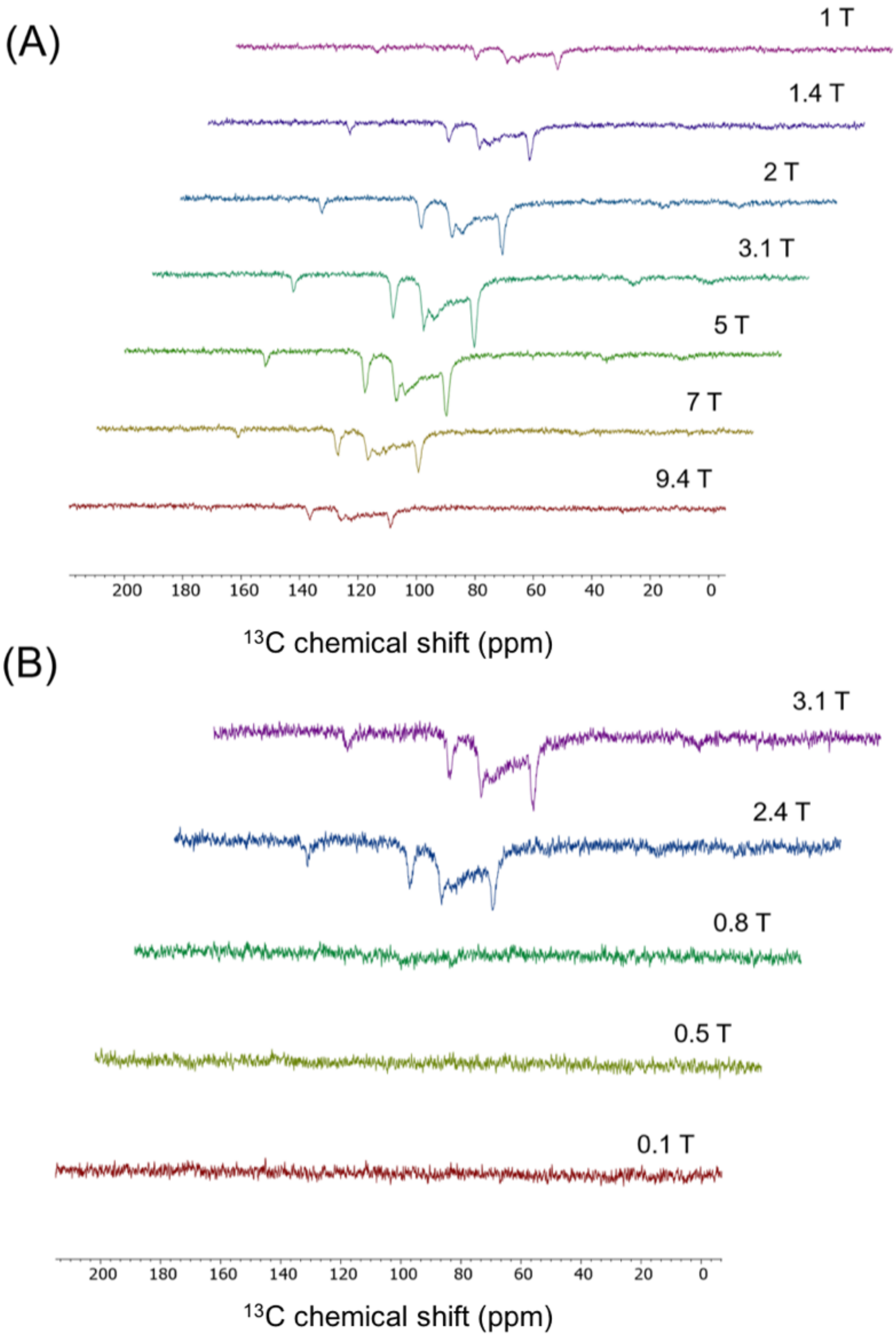

Figure S4. ^{13}C spectra of phototropin-LOV1-C57S measured under illumination at different magnetic fields in two groups (A) and (B) using the field-cycling system with 40 scans recorded at each magnetic field. Line

broadening was set to 10 Hz; the signal at ~110 ppm was integrated and referenced to that of 3.1 T measured in the corresponding experiment; the results of ^{13}C were plotted as intensity against the magnetic field in Figure 5.

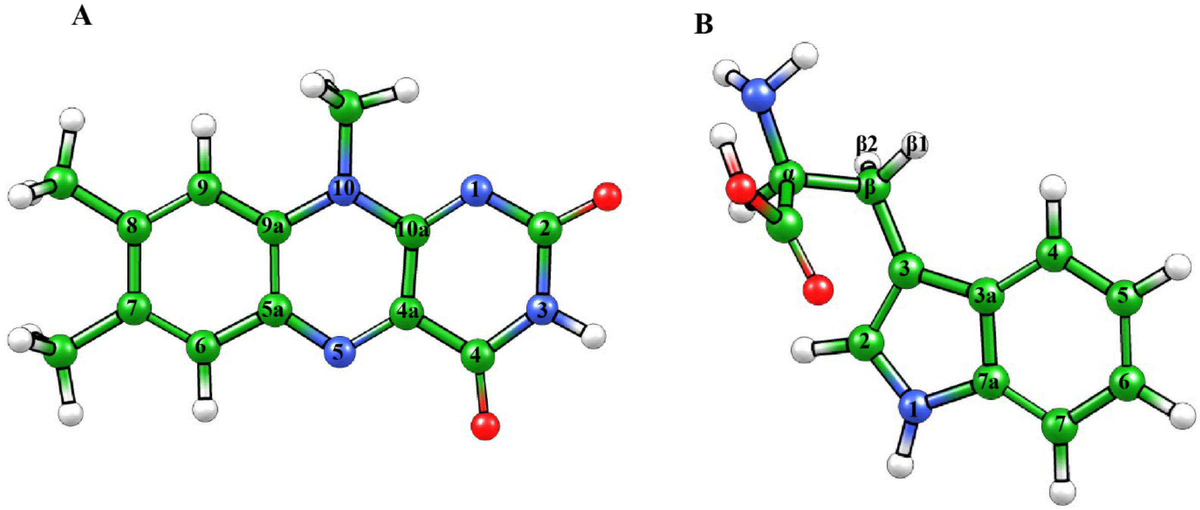

Figure S5. Optimized geometry of Lumi$^{\bullet-}$ (A) and TrpH$^{\bullet+}$ (B) with the numbering scheme for all magnetic nuclei.

Table S1. HFC tensors of Hβ1, C3 and N1 nuclei from the tryptophan in the PAS system taken from quantum chemical calculations.

HFC tensor of ^{1}H (Hβ1) in the PAS system [mT]	$A_{aa} = -0.08;\ A_{bb} = -0.04;\ A_{cc} = 0.12;$
HFC tensor of ^{13}C (C-3 of tryptophan) in the PAS system [mT]	$A_{aa} = -1.05;\ A_{bb} = -0.98;\ A_{cc} = 2.03;$
HFC tensor of ^{15}N (indole) in the PAS system [mT]	$A_{aa} = -0.2;\ A_{bb} = -0.18;\ A_{cc} = 0.38;$

Table S2. Parameters used for the theoretical calculation.

HYPERFINE INTERACTIONS

Value	Reference	Explanation
$a_{C_1} = 1.25$ mT<br>$a_{N_1} = 0.2$ mT	Alexey S. Kiryutin et al. *Supporting information*, J. Phys. Chem. B **111** (2007) 11221-11227.	The isotropic HFI constants are taken from that reference.
$a_{C_2} = 1$ mT<br>$a_{N_2} = -0.1$ mT<br>$a_{H_1} = 0.7$ mT<br>$a_{H_2} = 0.5$ mT	Alexey S. Kiryutin et al. *Supporting information*, J. Phys. Chem. B **111** (2007) 11221-11227.	These isotropic couplings are chosen to give the best fit to the experimental data. Nonetheless, the fitting parameters are of the same order of magnitude as HFC parameters presented in reference.
$b_{H_1} = 0.07$ mT<br>$b_{H_2} = 0.05$ mT<br>$b_{C_1} = 2.5$ mT<br>$b_{C_2} = 2$ mT<br>$b_{N_1} = 0.54$ mT<br>$b_{N_2} = -0.27$ mT	Present work: Table S1 in the Supporting information.	Pseudo-secular couplings b_i are estimated from quantum chemical calculations as differences between the principal values of the corresponding HFC tensors.

ZEEMAN INTERACTION AND ELECTRON-ELECTRON INTERACTION

Value	Reference	Explanation
$g_1 = 2.0034$	Benita Kopka et al., Sci. Rep. **7** (2017) 13346.	
$g_2 = 2.0026$	Ian Davis et al., J. Phys. Chem. A **122** (2018), 3170-3176, and Rebecca Pogni et al., J. Biol. Chem. **281** (2006) 9517-9526.	
$J_{ex} = -0.54$ mT		This value is chosen to give the best fit to the experimental data for ^{1}H-nucleus. The ^{1}H-nucleus is taken as reference, because CIDNP of ^{1}H is largely due to the anisotropic TSM mechanism (insofar as $\lvert\Delta\omega_e\rvert < \lvert\omega_N\rvert$).

REACTION RATES

Value	Reference	Explanation
$k_S = 0.01\, ns^{-1}$<br>$k_T = 0$		We assume that the SCRP is formed from the molecular triplet state, evolves into spin-correlated singlet state and recombines to the electronic ground state. k_T is taken as 0 since we considered triplet recombination to be very unlikely. Because of its very low photostationary concentration, we assume a fast decay of the SCRP of $\sim 100\, ns$ (therefore, k_S is taken as

		$0.01\ ns^{-1}$).
$k_{sc} =\ 0.001\ ns^{-1}$	Denis V. Sosnovsky et al., J. Chem. Phys. **144** (2016) 144202.	The scavenging rate is denoted as k_{sc}. This parameter is associated with the inverse lifetime of the SCRP. In liquids (where level crossings, LCs, are of great relevance), $k_{sc} = 1/\tau$, where τ is the time of diffusional separation of the radicals. The widths of the LC-derived features of CIDNP is sensitive to the lifetime of the SCRP. In solids (where level anti-crossings, LACs, are of great relevance) the width has a less pronounced dependence on k_{sc}. Since (i) we imply that the photo-CIDNP effect occurring in this cysteine-lacking LOV domain is the result of solid-state mechanisms even though it has been measured in solution state and since (ii) we compare the results of simulation in isotropic and anisotropic case, for the sake of generality, we assume the presence of a spin-independent decay of the SCRP with rate k_{sc} using the same value as in that reference.

DISTANCE

Value	References	Explanation
$\sim 1.6\ nm$	Sean Crosson & Keith Moffat, Plant Cell **14** (2002), 1067–1075 **(PDB 1N9L, 1N9N, and 1N9O)** and Roman Fedorov et al., Biophys. J. 84 (2003), 2474–2482 **(PDB 1JNU)**.	The distance between the two radical centers of Trp and FMN is given in the x-ray structures.
